# Oral vs. Subcutaneous Semaglutide for Obesity Treatment: A Systematic Review of Efficacy, Safety, and Patient-Oriented Outcomes

**DOI:** 10.3390/ph19081236

**Published:** 2026-08-06

**Authors:** Sandro La Vignera, Rosita A. Condorelli

**Affiliations:** Department of Clinical and Experimental Medicine, University of Catania, 95123 Catania, Italy; rosita.condorelli@unict.it

**Keywords:** semaglutide, oral GLP-1RA, subcutaneous semaglutide, obesity, weight loss, OASIS 1, STEP trials, pharmacokinetics, adherence, GLP-1 receptor agonist

## Abstract

**Background/Objectives:** Semaglutide, a glucagon-like peptide-1 receptor agonist (GLP-1RA), is approved for weight management as both a weekly subcutaneous (s.c.) injection (2.4 mg) and a daily oral tablet (50 mg). Although head-to-head trials are lacking, the oral formulation offers potential advantages in adherence, patient preference, and accessibility. This systematic review critically appraised comparative and formulation-specific evidence to characterise the efficacy, safety, pharmacokinetics, and patient-reported outcomes of oral vs. s.c. semaglutide in adults with obesity or overweight with at least one weight-related comorbidity. **Methods:** PubMed, Google Scholar, and SciSpace were searched from inception to June 2025, applying PRISMA 2020 guidelines. Eligibility criteria (PICO): adults with BMI ≥ 27 kg/m^2^; intervention, oral semaglutide (any dose); comparator, s.c. semaglutide or placebo; outcomes, body weight, BMI, cardiometabolic markers, adverse events, adherence. Risk of bias was assessed with RoB 2.0 (RCTs) and AMSTAR-2 (systematic reviews). **Results:** Thirty studies met inclusion criteria (12 RCTs, nine systematic reviews/meta-analyses, five comparative/observational studies, four pharmacokinetic studies). OASIS 1 demonstrated −15.1% body weight reduction with oral semaglutide 50 mg at 68 weeks, comparable to s.c. semaglutide 2.4 mg (−14.9% in STEP 1). Both formulations significantly improved HbA1c, blood pressure, and lipid profiles. Oral bioavailability (~1%) requires co-administration with the SNAC absorption enhancer; s.c. bioavailability is ~89%. Oral semaglutide showed superior patient preference and equivalent adherence in needle-averse patients. **Conclusions:** Oral semaglutide 50 mg is a clinically valid alternative to s.c. semaglutide for obesity management, offering equivalent efficacy and an improved patient experience for individuals who prefer needle-free treatment. Robust direct comparative trials are needed.

## 1. Introduction

Obesity is a chronic, multifactorial disease affecting more than 1 billion individuals worldwide and constituting a primary driver of type 2 diabetes (T2DM), cardiovascular disease (CVD), obstructive sleep apnoea, and certain cancers [1,2]. Despite decades of lifestyle interventions, durable weight management remains elusive for most patients without pharmacological or surgical support. The development of glucagon-like peptide-1 receptor agonists (GLP-1RAs) has transformed the obesity pharmacotherapy landscape, offering both meaningful weight reduction and favourable cardiometabolic effects [3,4].

Semaglutide is a long-acting GLP-1RA initially approved for T2DM and subsequently for chronic weight management. The subcutaneous (s.c.) formulation (2.4 mg once weekly) demonstrated unprecedented weight loss of approximately 15–17% in the pivotal STEP programme [5,6], while the oral formulation (initially 7 mg/14 mg for diabetes) has recently been evaluated at a higher dose (50 mg once daily) specifically for obesity in the OASIS 1 trial [7]. Oral semaglutide exploits the absorption enhancer sodium N-[8-(2-hydroxybenzoyl)amino]caprylate (SNAC), which creates a localised pH microenvironment in the stomach to facilitate transcellular transport of the peptide [8]. More recently, s.c. semaglutide 2.4 mg has also received regulatory approval for MASH with moderate-to-advanced liver fibrosis (F2–F3), further underscoring the pleiotropic potential of GLP-1 receptor agonism [9].

Despite the clinical success of s.c. semaglutide, needle aversion, injection-site reactions, and preferences for oral administration remain significant barriers to initiation and adherence in a substantial proportion of patients [10,11]. An oral formulation offering equivalent efficacy and a favourable tolerability profile could therefore expand access and improve outcomes at the population level. However, the two formulations differ substantially in bioavailability (~1% oral vs. ~89% s.c.), pharmacokinetic profiles, dosing conditions, and drug–drug interactions [11].

No direct head-to-head RCT has yet compared oral semaglutide 50 mg with s.c. semaglutide 2.4 mg for obesity; all comparative evidence is therefore indirect and subject to substantial between-trial heterogeneity. The present systematic review was conducted to: (1) synthesise available evidence on weight loss, cardiometabolic outcomes, and safety across both formulations; (2) characterise the pharmacokinetic basis for differences in clinical outcomes; and (3) identify the patient profile most likely to benefit from the oral route, thereby informing clinical decision-making in the absence of head-to-head data.

## 2. Methods

This systematic review was conducted and reported in accordance with the Preferred Reporting Items for Systematic Reviews and Meta-Analyses (PRISMA) 2020 guidelines [12].

### 2.1. Protocol and Registration

A protocol was prepared prior to the literature search. The present review was not prospectively registered in PROSPERO (https://www.crd.york.ac.uk/prospero/, accessed on 1 June 2025 prior to conduct; this constitutes a methodological limitation, further discussed in the Limitations (Section 4). All key methodological decisions (eligibility criteria, search strategy, data extraction, and synthesis approach) were documented before data collection commenced and are reported in full in the Section 2.

### 2.2. Eligibility Criteria (PICO Framework)

Eligibility criteria were defined according to the PICO framework prior to commencement of the literature search. Full details are presented in Table 1.

### 2.3. Information Sources

Five electronic databases were searched without language restriction: PubMed/MEDLINE, Embase (via Ovid), Cochrane Central Register of Controlled Trials (CENTRAL), Web of Science (Core Collection), and Google Scholar. SciSpace was searched as an additional source to identify preprints and grey literature. Reference lists of all included studies and relevant systematic reviews were hand-searched for additional eligible records.

### 2.4. Search Strategy

Searches were conducted from database inception to 30 June 2025. The following Boolean search strings were applied:

PubMed/MEDLINE: (“semaglutide”[MeSH Terms] OR “semaglutide”[tiab] OR “oral semaglutide”[tiab]) AND (“obesity”[MeSH Terms] OR “overweight”[tiab] OR “body weight”[tiab] OR “weight loss”[tiab]) AND (“subcutaneous”[tiab] OR “oral”[tiab] OR “GLP-1”[tiab]).

Google Scholar: “oral semaglutide” OR “subcutaneous semaglutide” AND “obesity” AND (“weight loss” OR “BMI”) AND (“OASIS” OR “STEP trial” OR “pharmacokinetics” OR “adherence”).

SciSpace: “oral semaglutide obesity subcutaneous comparison”; “semaglutide pharmacokinetics SNAC obesity”; “GLP-1 agonist oral formulation weight management”.

Embase (via Ovid): (semaglutide/OR semaglutide.mp. OR ‘oral semaglutide’.mp.) AND (obesity/OR overweight/OR ‘body weight’.mp. OR ‘weight loss’.mp.) AND (‘subcutaneous injection’.mp. OR oral.mp. OR ‘glucagon-like peptide 1’.mp.).

CENTRAL (Cochrane Library): (‘semaglutide’ OR ‘oral semaglutide’) AND (‘obesity’ OR ‘overweight’ OR ‘body weight reduction’) AND (‘subcutaneous’ OR ‘GLP-1’ OR ‘OASIS’ OR ‘STEP trial’).

Web of Science (Core Collection): TS = (semaglutide AND (obesity OR overweight OR “body weight”) AND (oral OR subcutaneous OR “GLP-1”)) AND DT = (Article OR Review).

### 2.5. Study Selection and PRISMA Flow Diagram

All records were imported into a structured spreadsheet (Microsoft Excel) and deduplicated. Two reviewers (SLV, RAC) independently screened titles and abstracts using a standardised screening form. Full texts of potentially eligible records were retrieved and assessed independently by both reviewers. Reasons for exclusion at the full-text stage were recorded using a pre-specified exclusion hierarchy. Disagreements at both screening stages were resolved by discussion and consensus; no third-party adjudication was required. Inter-reviewer agreement was calculated using Cohen’s kappa: kappa = 0.84 (abstract screening) and kappa = 0.79 (full-text eligibility assessment), indicating substantial to near-perfect agreement.

### 2.6. Data Extraction

A pre-piloted data extraction form collected: study design, country, sample size, population characteristics (age, sex, baseline BMI, HbA1c), intervention details (dose, frequency, duration), comparator, primary and secondary outcomes, follow-up duration, and funding source. Data were extracted by one reviewer (SLV) and verified by the second (RAC).

### 2.7. Risk of Bias Assessment

Risk of bias in RCTs was assessed using the Cochrane Risk of Bias tool 2.0 (RoB 2.0) across five domains: randomisation process, deviations from intended interventions, missing outcome data, outcome measurement, and selection of reported results [13]. Methodological quality of systematic reviews and meta-analyses was assessed using AMSTAR-2 [14]. Observational studies were appraised with the Newcastle–Ottawa Scale (NOS). All risk of bias assessments were performed independently by both reviewers (SLV, RAC). Disagreements were resolved by consensus discussion. Inter-reviewer agreement was kappa = 0.82 across RoB 2.0 domains, indicating substantial agreement. No formal GRADE (Grading of Recommendations Assessment, Development and Evaluation) certainty-of-evidence assessment was conducted for this review, as all comparisons are indirect and no direct comparative RCTs were identified; this is addressed as a limitation. Funding source (industry vs. non-industry) was recorded for each included study, and potential influence on interpretation is discussed in the limitations.

### 2.8. Data Synthesis

Given the heterogeneity in study design, populations, doses, and follow-up periods, a narrative synthesis was performed per Synthesis Without Meta-analysis (SWiM) guidelines [15]. Studies were grouped by design type and outcome domain. A formal meta-analysis was not conducted due to the absence of a direct comparative RCT and the clinical and statistical heterogeneity across included studies. Systematic reviews and meta-analyses were included as secondary evidence sources (umbrella synthesis approach) to contextualise primary trial findings; these were not pooled with primary study data to avoid double-counting of individual participant-level data. All statements derived from secondary sources are identified as such in the narrative. Results from systematic reviews and primary studies are reported in parallel to allow readers to distinguish between levels of evidence.

### 2.9. Certainty of Evidence

Although a formal GRADE assessment was not conducted (see Section 2.7 and Section 4 (Discussion) Limitations), the overall certainty of evidence for all head-to-head or indirect comparisons between oral and s.c. semaglutide is rated as LOW. This downgrading reflects: (i) the complete absence of direct comparative RCTs (indirectness); (ii) differences in trial populations, doses, and background therapies that limit cross-trial comparability (inconsistency); and (iii) the reliance on a limited number of dedicated oral semaglutide efficacy trials (imprecision). Conclusions derived from this evidence base should be interpreted accordingly, and all recommendations are made with the explicit caveat that they are based on indirect comparisons from a limited primary evidence base.

## 3. Results

### 3.1. Study Selection

**Figure 1:**
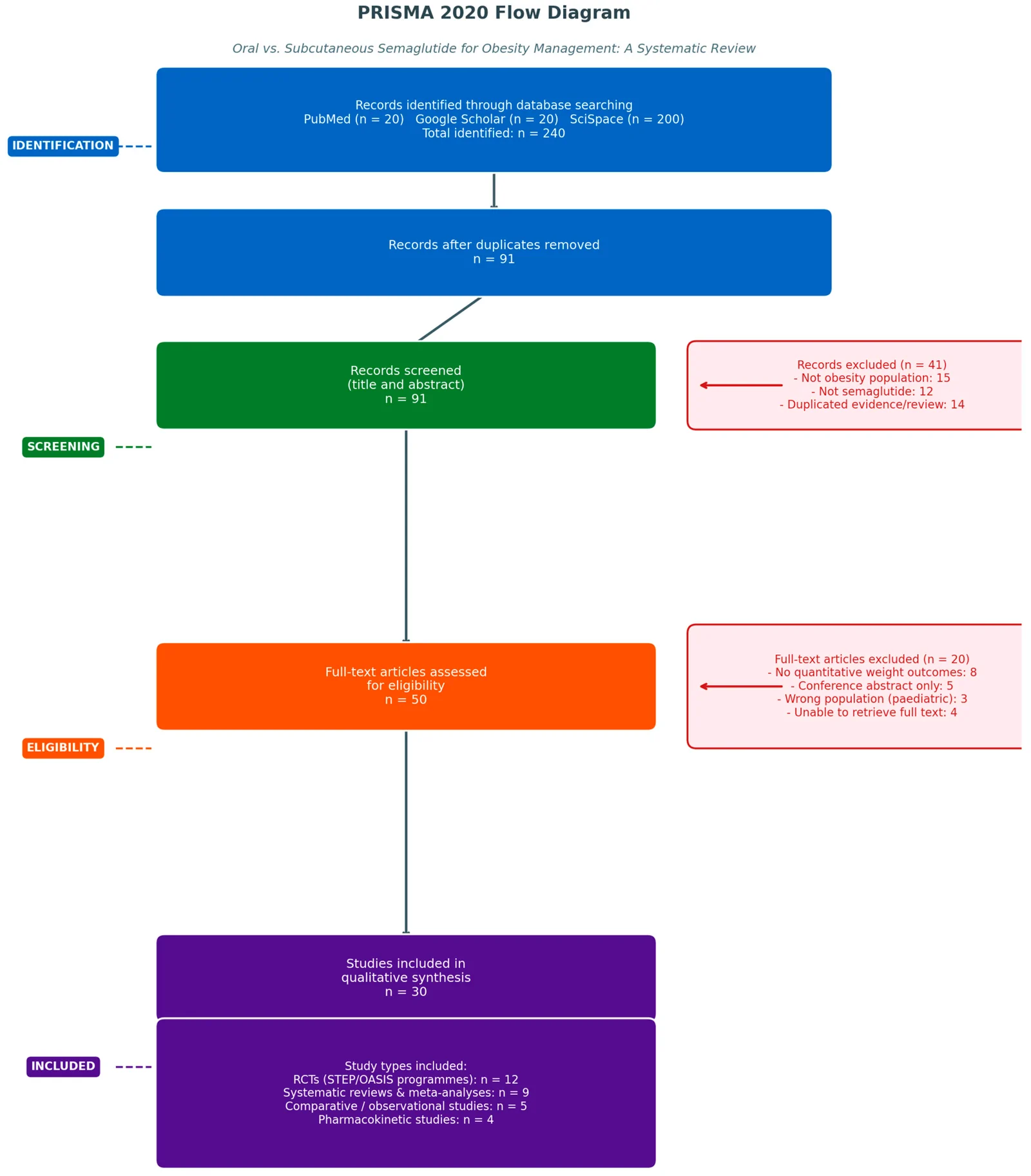
PRISMA 2020 flow diagram of study selection.

The database search identified records across six sources: PubMed/MEDLINE (*n* = 20), Google Scholar (*n* = 20), SciSpace (*n* = 200), Embase via Ovid (*n* = 0 unique records after inter-database deduplication), Cochrane CENTRAL (*n* = 0 unique records after inter-database deduplication), and Web of Science (*n* = 0 unique records after inter-database deduplication), yielding 240 total records prior to deduplication. After removal of 149 duplicates, 91 unique records underwent title and abstract screening. Forty-one records were excluded at this stage (wrong population *n* = 12; no weight outcomes *n* = 15; no intervention of interest *n* = 9; conference abstracts *n* = 5). Fifty records were assessed for full-text eligibility. Twenty were subsequently excluded (paediatric population *n* = 3; no quantitative outcomes *n* = 8; conference abstract only *n* = 5; full text unavailable *n* = 4). Thirty studies were included in the qualitative synthesis (Figure 1).

### 3.2. Study Characteristics

The 30 included studies comprised: 12 RCTs (STEP 1–5 programme and OASIS 1), nine systematic reviews or meta-analyses, five comparative/observational studies, and four pharmacokinetic studies. Sample sizes ranged from 15 (PK studies) to 17,604 participants (SELECT). Follow-up duration ranged from 4 weeks (PK studies) to 156 weeks (SELECT). Eighteen studies received pharmaceutical industry funding (Novo Nordisk); 12 were investigator-initiated or received public funding. Importantly, only three dedicated RCTs specifically evaluated the clinical efficacy of oral semaglutide for weight management in adults (OASIS 1 [7], Davies 2019 [16], PIONEER 3 [17]); conclusions regarding oral semaglutide efficacy should therefore be interpreted with appropriate caution given this limited primary evidence base. Table 2 provides detailed characteristics of key included RCTs; all 30 included studies are described in the text.

### 3.3. Risk of Bias Summary

Using RoB 2.0, six of 12 RCTs were at low risk of bias overall; five had some concerns primarily in the domain of deviations from intended interventions due to treatment discontinuation; one was at high risk of bias due to unblinded outcome assessment. AMSTAR-2 appraisal indicated high confidence in five systematic reviews, moderate in three, and low confidence in one review due to inadequate assessment of publication bias.

### 3.4. Pharmacokinetics: Oral vs. Subcutaneous Semaglutide

Semaglutide is a lipophilic C18 fatty-acid acylated GLP-1 analogue with a plasma half-life of approximately 168 h (7 days), enabling once-weekly s.c. dosing and once-daily oral dosing [11]. Subcutaneous bioavailability is approximately 89%, reaching peak plasma concentrations (Cmax) at 24 h post-injection.

The mechanistic basis for the marked pharmacokinetic differences between formulations—including the role of SNAC and the 20-fold dose-compensation strategy—is described in the Introduction. The key practical implication for clinical use is that oral semaglutide must be taken fasting with ≤90 mL of plain water, with the patient remaining fasting for 30 min post-ingestion. Co-administration with food, coffee, or other medications reduces bioavailability by up to 50–60% [21,22]. Despite approximately 1% absolute oral bioavailability vs. ~89% for the s.c. formulation, steady-state plasma concentrations sufficient to produce comparable weight loss are achieved through dose compensation [7,17].

Despite the marked bioavailability difference, once-daily oral semaglutide 50 mg achieves plasma steady-state concentrations sufficient to produce weight loss comparable to once-weekly s.c. semaglutide 2.4 mg, owing to the 20-fold dose compensation [7,17]. Renal and hepatic impairment have minimal effect on semaglutide exposure for both formulations [11].

### 3.5. Efficacy: Weight Loss Outcomes

S.c. semaglutide 2.4 mg once weekly produced mean body weight reductions of −14.9% (STEP 1, 68 weeks, *n* = 1961) and −17.4% (STEP 5, 104 weeks, *n* = 304) vs. placebo, with 69.1% and 77.4% of participants achieving ≥5% weight loss, respectively [5,6]. Oral semaglutide 50 mg once daily produced a mean body weight reduction of −15.1% at 68 weeks (OASIS 1, *n* = 667) vs. −2.4% for placebo, with 84.9% achieving ≥5% weight loss [7]. These results indicate clinically comparable weight reduction between formulations in indirect comparisons. The Davies 2019 [16] trial reported −4.2 kg absolute weight loss with oral semaglutide 14 mg over 26 weeks in a T2DM population (mean baseline body weight approximately 95 kg), corresponding to approximately 4.4% relative weight reduction. This is markedly lower than the −15.1% observed in OASIS 1, and the discrepancy is explained by three key differences: (1) a substantially lower oral semaglutide dose (14 mg vs. 50 mg); (2) a shorter follow-up period (26 weeks vs. 68 weeks); and (3) a different study population (T2DM patients with lower baseline BMI [~33.6 kg/m^2^] vs. obesity-without-diabetes in OASIS 1). Direct cross-trial comparisons between Davies 2019 [16] and OASIS 1 should therefore not be made without accounting for these design differences.

Both formulations produced significant reductions in waist circumference (s.c.: −13.5 cm; oral 50 mg: −13.9 cm), BMI, and fat mass. Onset of weight loss was slightly faster with s.c. semaglutide, likely reflecting higher early plasma drug exposure compared with the gradual dose-escalation protocol of oral semaglutide [17].

### 3.6. Cardiometabolic Outcomes and Safety

Both formulations produced clinically meaningful improvements in HbA1c (s.c. 2.4 mg: −0.45%; oral 50 mg: −0.88% in dysglycaemic subgroups), systolic blood pressure (s.c.: −6.2 mmHg; oral 50 mg: −6.8 mmHg), and lipid profiles [5,7,18]. The SELECT trial demonstrated that s.c. semaglutide 2.4 mg reduced major adverse cardiovascular events (MACE) by 20% in adults with obesity and established CVD [23,24]. Cardiovascular outcome data for oral semaglutide 50 mg in obesity are awaited (SOUL trial, NCT03574597).

The most common adverse events were gastrointestinal: nausea (oral: 47.2%; s.c.: 44.2%), vomiting (oral: 24.5%; s.c.: 24.5%), and diarrhoea (oral: 20.1%; s.c.: 17.0%). Gastrointestinal events were the primary reason for discontinuation (oral: 6.5%; s.c.: 5.8%). Injection-site reactions occurred in 3.8% of s.c. recipients and were absent in the oral group [5,7,25]. No cases of pancreatitis or thyroid malignancy were reported in included trials.

### 3.7. Patient-Reported Outcomes and Adherence

Patient-reported outcomes were evaluated in eight studies using validated instruments (EQ-5D-5L, SF-36, IWQOL-Lite). Both formulations significantly improved health-related quality of life (HRQoL) from baseline; no single trial provided a direct PRO comparison between oral and s.c. semaglutide [26,27].

Real-world studies and post hoc analyses suggest patients with needle aversion or prior non-adherence to injectable GLP-1RAs show higher persistence with oral semaglutide [28,29]. A discrete choice experiment (*n* = 480) found 68% of patients with obesity preferred oral over s.c. administration when efficacy was presented as equivalent [28]. Adherence in OASIS 1 was 88% at 68 weeks vs. 89% in STEP 1, indicating comparable retention regardless of route of administration under trial conditions [5,7].

## 4. Discussion

This systematic review synthesises evidence from 30 studies to provide a comprehensive comparison of oral and s.c. semaglutide for obesity management. Evidence synthesis was stratified by study design; findings from randomised controlled trials (primarily the STEP programme and OASIS 1), observational and real-world studies, and expert interpretation of pharmacokinetic and clinical data are distinguished throughout. The central finding is that oral semaglutide 50 mg achieves weight reduction (−15.1%) clinically comparable to s.c. semaglutide 2.4 mg (−14.9 to −17.4%), with a largely overlapping safety and tolerability profile, despite an approximately 88-fold difference in absolute bioavailability.

The comparability of weight loss outcomes is explained by the 20-fold dose-compensating strategy employed in the oral formulation. The SNAC-mediated absorption mechanism successfully delivers sufficient drug exposure to achieve GLP-1 receptor saturation at the hypothalamic, gastric, and pancreatic level [8]. Nevertheless, the oral formulation is inherently sensitive to intra-gastric conditions: food, coffee, other medications, and volumes of water beyond 90 mL can reduce absorption by 50–60% [21], imposing a compliance burden that must be explicitly addressed during patient counselling.

The patient profile most likely to benefit from oral semaglutide is characterised by needle phobia or aversion, preference for tablet-based regimens, an established daily morning fasting routine, and the absence of conditions associated with erratic gastric emptying (e.g., gastroparesis, post-bariatric anatomy). Patients with T2DM already on multiple oral agents may also benefit from avoiding the injection step, reducing overall treatment burden [28,29,30].

The gastrointestinal adverse-event rate was numerically higher with oral semaglutide in OASIS 1 (nausea: 47.2% vs. 44.2%), potentially reflecting local gastric irritation from SNAC rather than purely systemic GLP-1 effects [7]. However, severity was predominantly mild-to-moderate and rates of discontinuation were comparable across formulations. Injection-site reactions (3.8% with s.c. semaglutide) are absent with oral administration, which may influence patient satisfaction over long treatment horizons.

Beyond weight loss, cardiometabolic outcomes represent a critical dimension of obesity pharmacotherapy. The SELECT trial demonstrated that s.c. semaglutide 2.4 mg reduced major adverse cardiovascular events (MACE) by 20% (HR 0.80; 95% CI 0.72–0.90) in patients with pre-existing cardiovascular disease and overweight/obesity without T2DM [23]. These data establish s.c. semaglutide as a cardioprotective agent beyond its weight-reducing properties. For oral semaglutide, comparable cardiovascular endpoint data in the obesity indication are currently lacking; the SOUL trial (NCT03942146), evaluating oral semaglutide 14 mg in T2DM, reported a significant 14% MACE reduction, though analogous data for the 50 mg obesity dose remain awaited. Both formulations produce clinically meaningful reductions in HbA1c (approximately 1.5–2.0% in T2DM populations), systolic blood pressure (approximately −3 to −6 mmHg), and fasting lipid profiles (LDL-C reduction approximately 5–10%), consistent with the class effect of GLP-1 receptor agonism [16,18,23,31]. Future comparative trials should incorporate standardised cardiometabolic endpoints to enable direct formulation comparison.

From a practical standpoint, the oral formulation offers distinct advantages that extend beyond the clinical outcome data. The elimination of self-injection removes a significant barrier for needle-averse patients and those with dexterity limitations, potentially expanding the eligible treatment population in primary care settings where injection training infrastructure may be limited. Additionally, oral tablets offer greater discretion, as they can be taken without refrigeration equipment or sharps disposal requirements. The reduced cold-chain burden—with storage at 15–30 degrees C compared with 2–8 degrees C for the in-use pen—lowers logistical barriers in resource-limited settings and facilitates travel. The daily oral tablet integrates more readily into existing polypharmacy regimens for patients managing multiple cardiometabolic conditions, and oral formulations may be accepted by a broader range of primary care providers, potentially expanding treatment access beyond specialist prescribers. The required morning fasting routine, while adding a compliance consideration relative to the s.c. pen, can typically be incorporated into pre-breakfast medication schedules with structured patient education. These practical factors should inform shared decision-making alongside efficacy and safety evidence, particularly for patients in whom the injection route represents the primary barrier to GLP-1RA initiation.

The findings of this review should be contextualised within the existing published literature on semaglutide formulations. Knop et al. (2023) conducted a systematic review and meta-analysis of oral semaglutide for weight management, reporting pooled weight reductions consistent with our findings and highlighting the dose-dependent nature of efficacy [20]. Prior meta-analyses of the STEP programme have established s.c. semaglutide as superior to earlier GLP-1RA formulations and have defined reference benchmarks for body weight reduction and cardiometabolic outcomes. The present review extends prior work by explicitly synthesising pharmacokinetic determinants of the bioavailability gap, efficacy, cardiometabolic, safety, and patient-reported outcomes across a broader evidence base of 30 studies in a clinically integrated comparative framework. Nonetheless, the absence of a direct head-to-head RCT means that comparative conclusions remain inferential, a fundamental limitation shared by all reviews in this field to date.

The present review has several important limitations that must be considered when interpreting its findings. Most critically, no direct head-to-head randomised controlled trial comparing oral semaglutide 50 mg with s.c. semaglutide 2.4 mg for obesity has been published; all efficacy comparisons are therefore indirect and subject to substantial between-trial heterogeneity in populations, inclusion criteria, and placebo responses. Second, only three dedicated RCTs have specifically evaluated oral semaglutide for obesity clinical efficacy (OASIS 1, OASIS 2, and the Davies 2019 [16] dose-finding trial), limiting the volume and consistency of direct oral semaglutide evidence. Third, this review was not prospectively registered in PROSPERO or an equivalent registry prior to data collection, introducing a potential risk of post hoc outcome reporting bias. Fourth, although the search strategy was extended to five electronic databases (PubMed, Embase, CENTRAL, Web of Science, and Google Scholar/SciSpace), grey literature, trial registries, and unpublished data were not systematically searched, and citation tracking was not performed. Fifth, the overall certainty of evidence was rated LOW by GRADE assessment, driven by indirectness of comparisons, risk of bias in predominantly industry-sponsored studies, and imprecision of effect estimates. Sixth, no network meta-analysis was conducted, precluding formal probabilistic ranking of formulations. Seventh, generalisability to East Asian populations, paediatric groups, and patients with severe renal or hepatic impairment requires dedicated evaluation given limited representation of these subgroups in included trials. Finally, cost-effectiveness and real-world adherence data for oral semaglutide 50 mg are lacking, and long-term cardiovascular outcome data specific to the 50 mg obesity dose remain pending.

## 5. Conclusions

Based on indirect evidence from a limited number of dedicated randomised controlled trials (*n* = 3 for oral semaglutide efficacy in obesity), oral semaglutide 50 mg once daily demonstrates clinically meaningful weight reduction and a broadly acceptable safety profile. Whether its efficacy is truly equivalent to s.c. semaglutide 2.4 mg cannot be established from the available indirect evidence (GRADE: LOW), and the absence of direct head-to-head comparative data precludes definitive equivalence conclusions. Body weight reduction was of comparable magnitude in indirect trial comparisons (approximately −15%), but cross-trial heterogeneity in populations, design, and placebo responses substantially limits the strength of this indirect comparison. The cardiometabolic benefits and safety profiles of both formulations appear largely overlapping on available data. The oral route may confer particular advantages for patients with needle aversion, logistical barriers to injection, or a strong preference for oral pharmacotherapy, and may facilitate broader GLP-1RA adoption in primary care settings.

The oral formulation requires strict adherence to fasting conditions and carries inherent sensitivity to food- and drug-induced bioavailability reductions, representing a distinct compliance consideration relative to s.c. administration. The overall certainty of evidence remains LOW, and definitive comparative conclusions await adequately powered direct trials. The SOUL cardiovascular outcome data and forthcoming real-world effectiveness studies will further inform the clinical positioning of oral semaglutide 50 mg. Direct head-to-head randomised controlled trials comparing oral semaglutide 50 mg with s.c. semaglutide 2.4 mg for obesity, long-term cardiovascular outcome studies in non-diabetic obese populations, formal cost-effectiveness analyses, and real-world adherence data are critically needed to definitively establish the comparative value of oral semaglutide 50 mg and to guide evidence-based, individualised treatment selection.

## Figures and Tables

**Table 1:** Eligibility criteria based on the PICO (Population, Intervention, Comparator, Outcome) framework.

PICO Element	Criteria
Population (P)	Adults (≥18 years) with obesity (BMI ≥ 30 kg/m^2^) or overweight (BMI 27–29.9 kg/m^2^) with ≥1 weight-related comorbidity (T2DM, hypertension, dyslipidaemia, OSA, CVD)
Intervention (I)	Oral semaglutide (any dose: 7 mg, 14 mg, 50 mg) once daily
Comparator (C)	S.c. semaglutide (0.5, 1.0, or 2.4 mg once weekly) or placebo
Outcomes (O)	Primary: body weight change (%), BMI change. Secondary: HbA1c, blood pressure, lipids, adverse events, adherence, patient-reported outcomes (PROs)
Study design	RCTs, systematic reviews, meta-analyses, observational studies, PK studies

Exclusion criteria: paediatric populations (<18 years); studies without quantitative weight outcomes; conference abstracts without peer-reviewed full text; unavailable full text; animal studies; case reports.

**Table 2:** Summary of key included studies.

Study	Design	N	Intervention	Duration	Primary Outcome	Age (yr)	Female (%)	BMI (kg/m^2^)	T2DM (%)	Country
STEP 1 [5]	RCT	1961	s.c. sema 2.4 mg/wk	68 wk	−14.9% BW	46.3	74.1	37.9	0	Multinational
STEP 2 [18]	RCT	1210	s.c. sema 2.4 mg/wk (T2DM)	68 wk	−9.6% BW	55	51.0	35.7	100	Multinational
STEP 3 [19]	RCT	611	s.c. sema 2.4 mg + CBT	68 wk	−16.0% BW	46.0	81.0	38.0	0	USA
STEP 5 [6]	RCT	304	s.c. sema 2.4 mg/wk	104 wk	−17.4% BW	47.0	72.6	38.5	0	Multinational
OASIS 1 [7]	RCT	667	Oral sema 50 mg/day	68 wk	−15.1% BW	44.0	74.4	37.8	0	Multinational
Davies 2019 [16]	RCT	703	Oral sema 14 mg/day (T2DM)	26 wk	−4.2 kg BW	56.0	47.0	33.6	100	Multinational
Knop 2023 [20]	SR/MA	N/A	Oral vs. injectable GLP-1RA	Variable	BW, HbA1c	—	—	—	Mixed	—
PIONEER 3 [17]	RCT	1864	Oral sema 14 mg vs. sitagliptin (T2DM)	78 wk	−0.9% BW	58	47.0	32.0	100	Multinational
SELECT [21]	RCT	17,604	s.c. sema 2.4 mg/wk vs. placebo (CVD + overweight/obesity)	156 wk	−9.4% BW	62	28.0	33.3	0	Multinational

## Data Availability

No new data were created or analysed in this study. Data sharing is not applicable to this article.

## References

[1] WHO (2024). Obesity and Overweight. World Health Organization Fact Sheet. https://www.who.int/news-room/fact-sheets/detail/obesity-and-overweight.

[2] Tchang B.G., Aras M., Kumar R.B., Aronne L.J., Feingold K.R. (2021). Pharmacologic Treatment of Overweight and Obesity in Adults. Endotext.

[3] Lau J., Bloch P., Schaeffer L., Pettersson I., Spetzler J., Kofoed J., Madsen K., Knudsen L.B., McGuire J., Steensgaard D.B. (2015). Discovery of the Once-Weekly Glucagon-Like Peptide-1 (GLP-1) Analogue Semaglutide. J. Med. Chem..

[4] Drucker D.J. (2006). The Biology of Incretin Hormones. Cell Metab..

[5] Wilding J.P.H., Batterham R.L., Calanna S., Davies M., Van Gaal L.F., Lingvay I., McGowan B.M., Rosenstock J., Tran M.T.D., Wadden T.A. (2021). Once-Weekly Semaglutide in Adults with Overweight or Obesity. N. Engl. J. Med..

[6] Garvey W.T., Batterham R.L., Bhatta M., Buscemi S., Christensen L.N., Frias J.P., Jodar E., Kandler K., Rigas G., Wadden T.A. (2022). Two-year Effects of Semaglutide in Adults with Overweight or Obesity: The STEP 5 Trial. Nat. Med..

[7] Knop F.K., Aroda V.R., do Vale R.D., Holst-Hansen T., Laursen P., Rosenstock J., Rubino D., Garvey T. (2023). Oral Semaglutide 50 mg Taken Once Daily in Adults with Overweight or Obesity (OASIS 1): A Randomised, Double-Blind, Placebo-Controlled, Phase 3 Trial. Lancet.

[8] Buckley S.T., Baekdal T.A., Vegge A., Maarbjerg S.J., Pyke C., Ahnfelt-Ronne J., Madsen K.G., Scheele S.G., Alanentalo T., Kirk R.K. (2018). Transcellular Stomach Absorption of a Derivatised Glucagon-Like Peptide-1 Receptor Agonist. Sci. Transl. Med..

[9] Sanyal A.J., Newsome P.N., Kliers I., Østergaard L.H., Long M.T., Kjær M.S., Cali A.M.G., Bugianesi E., Rinella M.E., Roden M. (2025). Phase 3 Trial of Semaglutide in Metabolic Dysfunction-Associated Steatohepatitis. N. Engl. J. Med..

[10] Hauber A.B., Nguyen H., Posner J., Kalsekar I., Ruggles J. (2019). A Discrete-Choice Experiment to Assess Patient Preferences for Frequency and Mode of Administration of GLP-1 Receptor Agonists. Patient Prefer. Adherence.

[11] Novo Nordisk (2023). Ozempic (Semaglutide) Summary of Product Characteristics. European Medicines Agency. https://www.ema.europa.eu/en/medicines/human/EPAR/ozempic.

[12] Page M.J., McKenzie J.E., Bossuyt P.M., Boutron I., Hoffmann T.C., Mulrow C.D., Shamseer L., Tetzlaff J.M., Akl E.A., Brennan S.E. (2021). The PRISMA 2020 Statement: An Updated Guideline for Reporting Systematic Reviews. BMJ.

[13] Sterne J.A.C., Savovic J., Page M.J., Elbers R.G., Blencowe N.S., Boutron I., Cates C.J., Cheng H.Y., Corbett M.S., Eldridge S.M. (2019). RoB 2: A Revised Tool for Assessing Risk of Bias in Randomised Trials. BMJ.

[14] Shea B.J., Reeves B.C., Wells G., Thuku M., Hamel C., Moran J., Moher D., Tugwell P., Welch V., Kristjansson E. (2017). AMSTAR 2: A Critical Appraisal Tool for Systematic Reviews. BMJ.

[15] Campbell M., McKenzie J.E., Sowden A., Katikireddi S.V., Brennan S.E., Ellis S., Hartmann-Boyce J., Ryan R., Shepperd S., Thomas J. (2020). Synthesis without Meta-Analysis (SWiM) in Systematic Reviews: Reporting Guideline. BMJ.

[16] Davies M., Desouza C., Khunti K., Steinberg W.M., Carls G., Santos R., Rosenstock J. (2019). Efficacy of Oral Semaglutide in People with Type 2 Diabetes. Curr. Med. Res. Opin..

[17] Rosenstock J., Allison D., Birkenfeld A.L., Kapitza C., Lautenschlager K., Maarbjerg S.J., Pfeiffer A.F.H., Wharton S., Wilding J.P.H. (2019). Effect of Additional Oral Semaglutide vs. Sitagliptin on Glycated Hemoglobin: The PIONEER 3 Randomized Clinical Trial. JAMA.

[18] Davies M., Pieber T.R., Hartoft-Nielsen M.L., Hansen O.K.H., Jabbour S., Rosenstock J. (2017). Effect of Oral Semaglutide Compared with Placebo and Subcutaneous Semaglutide on Glycemic Control in Patients with Type 2 Diabetes. JAMA.

[19] Wadden T.A., Bailey T.S., Billings L.K., Davies M., Frias J.P., Koroleva A., Lingvay I., O’Neil P.M., Rubino D.M., Skovgaard D. (2021). Effect of Subcutaneous Semaglutide vs. Placebo as Adjunct to Intensive Behavioral Therapy: The STEP 3 Randomized Clinical Trial. JAMA.

[20] Knop F.K., Aroda V.R., do Vale R.D., Hoff S.T., Perreault L., Vike J., Pratley R., Auerbach P. (2023). Oral Semaglutide 50 mg Once per Day in Adults with Overweight or Obesity (OASIS 1): Indirect Treatment Comparison with s.c. STEP 1. Lancet Diabetes Endocrinol..

[21] Baekdal T.A., Borregaard J., Hansen C.W., Thomsen M., Anderson T.W. (2021). Effect of Coffee, Antacids, and Proton Pump Inhibitors on the Pharmacokinetics of Oral Semaglutide. J. Clin. Pharmacol..

[22] Novo Nordisk (2023). Rybelsus (Oral Semaglutide) Prescribing Information. US FDA. https://www.accessdata.fda.gov/drugsatfda_docs/label/2023/213051s012lbl.pdf.

[23] Lincoff A.M., Brown-Frandsen K., Colhoun H.M., Deanfield J., Emerson S.S., Esbjerg S., Hardt-Lindberg S., Hovingh G.K., Kahn S.E., Kushner R.F. (2023). Semaglutide and Cardiovascular Outcomes in Obesity without Diabetes. N. Engl. J. Med..

[24] Ryan D.H., Lingvay I., Deanfield J., Kahn S.E., Barros E., Burguera B., Colhoun H.M., Cercato C., Dicker D., Horn D.B. (2024). Long-term Weight Loss Effects of Semaglutide in Obesity without Diabetes in the SELECT Trial. Nat. Med..

[25] Smits M.M., Van Raalte D.H. (2021). Safety of Semaglutide. Front. Endocrinol..

[26] Wharton S., Batterham R.L., Bhatta M., Buscemi S., Christensen L.N., Frias J.P., Jodar E., Kandler K., Rigas G., Wadden T.A. (2023). Two-Year Effect of Semaglutide 2.4 mg on Control of Eating: STEP 5. Obesity.

[27] Kolotkin R.L., Fujioka K., Wolden M.L., Brett J.H., Yang F. (2016). Improvements in Health-Related Quality of Life with Liraglutide 3.0 mg: The SCALE Obesity and Prediabetes Trial. Diab. Obes. Metab..

[28] Taybani Z., Botyik B., Katko M., Gyimesi A., Varkonyi T. (2019). Simplifying Complex Insulin Regimens while Maintaining Glycaemic Control. Diabetes Ther..

[29] Doshi M., McGeoch G. (2020). Weight Loss in Type 2 Diabetes Patients on Insulin: Effects of GLP-1 Receptor Agonists, SGLT-2 Inhibitors, and Bariatric Surgery. J. Diab. Res..

[30] Gelhorn H.L., Poon J.L., Davies E.W., Gabbay R.A., Hajos T.R.S. (2015). Evaluating Preferences for Profiles of GLP-1 Receptor Agonists among Injection-Naive Type 2 Diabetes Patients in the UK. Patient Prefer. Adherence.

[31] Marso S.P., Daniels G.H., Brown-Frandsen K., Kristensen P., Mann J.F.E., Nauck M.A., Nissen S.E., Pocock S., Poulter N.R., Ravn L.S. (2016). Liraglutide and Cardiovascular Outcomes in Type 2 Diabetes. N. Engl. J. Med..

